# Constructing Benchmark Databases and Protocols for Medical Image Analysis: Diabetic Retinopathy

**DOI:** 10.1155/2013/368514

**Published:** 2013-06-19

**Authors:** Tomi Kauppi, Joni-Kristian Kämäräinen, Lasse Lensu, Valentina Kalesnykiene, Iiris Sorri, Hannu Uusitalo, Heikki Kälviäinen

**Affiliations:** ^1^Machine Vision and Pattern Recognition Laboratory, Department of Mathematics and Physics, Lappeenranta University of Technology (LUT), Skinnarilankatu 34, FI-53850 Lappeenranta, Finland; ^2^Department of Signal Processing, Tampere University of Technology, Korkeakoulunkatu 10, FI-33720 Tampere, Finland; ^3^Department of Ophthalmology, University of Eastern Finland, Yliopistonranta 1, FI-70211 Kuopio, Finland; ^4^Department of Ophthalmology, University of Tampere, Biokatu 14, FI-33520 Tampere, Finland

## Abstract

We address the performance evaluation practices for developing medical image analysis methods, in particular, how to establish and share databases of medical images with verified ground truth and solid evaluation protocols. Such databases support the development of better algorithms, execution of profound method comparisons, and, consequently, technology transfer from research laboratories to clinical practice. For this purpose, we propose a framework consisting of reusable methods and tools for the laborious task of constructing a benchmark database. We provide a software tool for medical image annotation helping to collect class label, spatial span, and expert's confidence on lesions and a method to appropriately combine the manual segmentations from multiple experts. The tool and all necessary functionality for method evaluation are provided as public software packages. As a case study, we utilized the framework and tools to establish the DiaRetDB1 V2.1 database for benchmarking diabetic retinopathy detection algorithms. The database contains a set of retinal images, ground truth based on information from multiple experts, and a baseline algorithm for the detection of retinopathy lesions.

## 1. Introduction

Image databases and expert ground truth are regularly used in medical image processing. However, it is relatively common that the data is not public, and, therefore, reliable comparisons and state-of-the-art surveys are difficult to conduct. In contrast to, for example, biometrics including face, iris, and fingerprint recognition, the research has been driven by public databases and solid evaluation protocols. These databases have been extended and revised resulting in continuous pressure for the development of better methods. For every medical application, it should be an acknowledged scientific contribution to provide a set of images, collect accurate and reliable ground truth for the images, and devise a meaningful evaluation protocol. Once this pioneering work has been done, it sets an evaluation standard for a selected problem.

We have set our primary goal to the automatic detection of diabetic retinopathy [1] which is very well motivated since diabetes has become one of the most rapidly increasing health threats worldwide [2, 3]. Since the retina is vulnerable to microvascular changes of diabetes and diabetic retinopathy is the most common complication of diabetes, retinal imaging is considered a noninvasive and painless mean to screen and monitor the progress of the disease [4]. Since these diagnostic procedures as well as regular monitoring of state of diabetes require the attention of medical personnel, for example, GP and ophthalmologists, the workload and shortage of personnel will eventually exceed the current resources for screening. To cope with these challenges, digital imaging of the eye fundus, and automatic or semiautomatic image analysis algorithms based on image processing and computer vision techniques provide a great potential. For this, suitable retinal image databases containing well-defined and annotated ground truth are needed.

**Figure 1:**
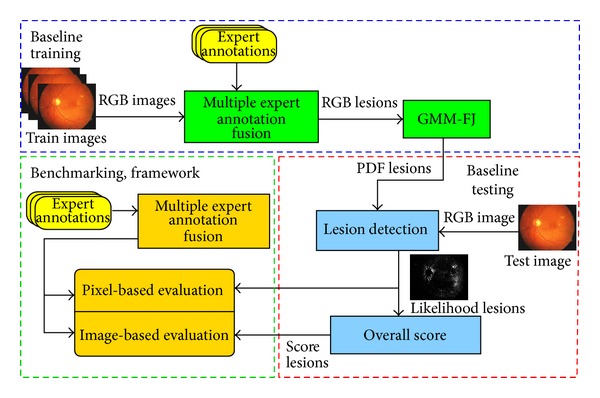
A framework for constructing benchmark databases and protocols [1].

In this work, our main contributions are (1) an image annotation tool for medical experts, (2) a public retinal image database with expert annotations, (3) a solid evaluation framework for the image analysis system development and comparison (Figure 1), and (4) image-based and pixel-based evaluation methods. We particularly focus on constructing benchmark databases and protocols. We have experienced that developing databases from scratch is demanding, laborious, and time consuming. However, certain tasks occur repeatedly and are reusable as such. Here, we discuss the related practical issues, point out and solve repeated occurring subtasks, and provide the solutions as open-source tools on our website. In the experimental part, we utilize the proposed framework and construct a revised version of the diabetic retinopathy database DiaRetDB1 originally published in [5, 6], and later discussed in [7]. 

The paper is organized as follows: in Section 2, we discuss medical benchmarking in general, provide relevant guidelines, and briefly survey the related works. In Section 3, we discuss collecting patient images and the spatial ground truth. We propose a portable data format for the ground truth, and represent and solve the problem of fusing multiple expert annotations. In Section 4, we discuss evaluation practices in general, and provide an evaluation approach based on the standard ROC analysis. We evaluate our color-cue-based detection method (baseline) by using the constructed database. In Section 5, we utilize the given results and tools to establish the diabetic retinopathy evaluation and benchmarking database DiaRetDB1 V2.1, and we draw the conclusions in Section 6.

## 2. Benchmarking in General and Previous Work

Public image databases for benchmarking purposes are essential resources in the development of image analysis algorithms and help medical imaging researchers evaluate and compare state-of-the-art methods. Eventually, this leads to the development of better algorithms and, consequently, will support technology transfer from research laboratories to clinical practice. However, the public availability of image databases is limited because of the amount of work needed to make internal data publicly available, including the ground truth annotation and the privacy protection of the patient information. Therefore, reliable comparisons and state-of-the-art surveys are difficult to perform. In this section, a benchmarking framework is described that provides guidelines on how to construct benchmarking image databases with a particular emphasis on retinal image analysis. The benchmarking framework comprises three important requirements: (1) patient images, (2) the ground truth, and (3) an evaluation protocol.

### 2.1. Key Questions in Constructing Benchmarks

Thacker et al. [10] studied the performance characterization of computer vision methods. They provide good examples which are easily transferable to applications of medical image processing. The results in [10] can be utilized in every step of the method development, but we set special attention to the final diagnosis, that is, the subject-wise decision making directly serving the clinical work. In other words, the framework omits the development and research phase evaluations and constructs the good practices to evaluate the performance of retinal image analysis algorithms. For that purpose, the eight general considerations adopted from [10] are addressed and referred to as the key questions.

C1: “How is testing currently performed?” If a commonly used database and protocol are available, their validity for the development and evaluation needs to be examined. In the worst case, a new database needs to be constructed for which the proposed framework can be useful.
C2: “Is there a data set for which the correct answers are known?” Such a data set can be used to report the results in accordance to other studies. This enables method comparison.
C3: “Are there data sets in common use?” See C1 and C2. Common data sets facilitate fair method comparison.
C4: “Are there experiments which show that algorithms are stable and work as expected?” These experiments can be realized if representative data and expert ground truth are available.
C5: “Are there any strawman algorithms?” If a strawman algorithm is included in the database, it defines the baseline performance for other methods. In this paper, we call these kinds of baseline methods as strawman algorithms.
C6: “What code and data are available?” By publishing the method's code or at least executable version of it, other research groups can avoid laborious reimplementation.
C7: “Is there a quantitative methodology for the design of algorithms?” This depends on the medical problem, but the methodology can be typically devised by following corresponding clinical work and practices. Understanding of the medical practitioners' task which should be assisted or automated provides a conceptual guideline. If the database is correctly built to reflect the real-world conditions, then the database implicitly reflects the applicability of the algorithm's design to the problem.
C8: “What should we be measuring to quantify performance? which metrics are used?” At least in the image-wise (subject-wise) experiments, the receiver operating characteristic (ROC) curve is in accordance with the medical practice, where the sensitivity and specificity values are in common use. The ROC curve, also known as ROC analysis, is a widely used tool in medical community for visualizing and comparing methods based on their performance [11]. It is a graphical representation that describes the trade-off between the sensitivity and specificity (e.g., correctly classified normal images versus correctly classified abnormal images). In the curve, the *x*-axis is defined as 1 − specificity, and the *y*-axis is directly the sensitivity [12].

In general, C1 ∈ C2 ∈ C3, which means that if there is a commonly used data set in the form of, for example, a benchmark database, the answers to C1 and C2 are known. Similarly, C4 ∈ C5 ∈ C6 defines the maturity of the existing solutions. In the case where the data and code are both available and have been shown to work by achieving the required sensitivity and specificity rates, the solution is at a mature level and true clinical experiments can be started. C7 is a general guideline for the design to find an acceptable work flow for a specific problem, and C8 sets the quantitative and meaningful performance measures.

### 2.2. Requirements for Benchmarking

Benchmarking image databases in retinal imaging require three mandatory components: (1) patient images, (2) ground truth by domain experts, and (3) an evaluation protocol. Additional components, such as a baseline algorithm, provide notable additional value, but in the following, the three mandatory components are discussed.

#### 2.2.1. True Patient Images

True patient images carry information which is meaningful for solving a given problem; that is, algorithms which work with these images are expected to perform well also in practice. The images can be recorded using alternative subjects, such as animals that are physiologically close to humans, and disease-related lesions can be produced artificially by using various substances. These are standard practices in medical research, but before drawing any general conclusions, their relevance and accuracy to the real world must be carefully verified. With true patient images, the results are biased by the distribution of database images with respect to the specific real population. The collection and selection of images are further discussed in Section 3. The true patient image requirement concerns the key questions C2, C3, C4, and C6.

#### 2.2.2. Ground Truth Given by Experts

Ground truth must be accurate and reliable in the sense that it is statistically representative over experts. In the field of retinal image processing, it is advisable that the tools for ground truth annotation are provided by computer vision scientists, but the images are selected and annotated by medical experts specialized in the field. It is also clear that the ground truth must be independently collected from multiple experts. This can be laborious and expensive, but it enables statistical studies of reliability. In the case of multiple experts, disambiguation of the data is often necessary prior to the application of machine learning methods. Collecting the ground truth from experts concerns the key questions C2, C3, C4, and C6.

#### 2.2.3. Evaluation Protocol

A valid evaluation protocol providing quantitative and comparable information is essential for reliable performance evaluations. Most articles related to retinal image analysis report the sensitivity and specificity separately, but they are meaningless metrics unless a method can produce superior values for both. The golden standard in similar problems is the ROC analysis. The approach is essentially the same as reporting the sensitivity and specificity but provides the evaluation result over all possible combinations of these values. It turns out that in benchmarking, the comparison of ROC curves is problematic, and, therefore, specific well-justified operation points or the area under curve (AUC) can be used as a single measure. This issue is further discussed in Section 4. In addition to the evaluation protocol, a baseline method (C5) or at least the results with the baseline method are helpful since they set the performance level which new methods should clearly outperform. From another viewpoint, the best reported results by using a commonly accepted database set the state of the art. The evaluation protocol requirement concerns the key questions C1, C4, C7, and C8.

### 2.3. Eye Disease Databases

This section describes the most important public benchmarking databases in retinal image analysis. The database review provides a short description for each database, where the key questions C1–C8 addressed in Section 2.1 are used to highlight the main properties. Since each database is publicly available, they are expected to be in common use (C3). See Table 1 for a short summary.

STARE (structured analysis of the retina) [17] is one of the most used reference image database in the literature (C3, C4) for comparing blood vessel detection and optic disc localization algorithms. The STARE website [17] provides 20 images with pixel-wise hand-labeled ground truth for blood vessel detection (C2) and 81 images for optic disc localization without ground truth. The performance of blood vessel detection is measured using the ROC curve analysis, where the sensitivity is the proportion of correctly classified blood vessel pixels and the specificity is the proportion of correctly classified normal pixels (C8.1) [18]. In the evaluation of optic disc localization, the proportion of correctly localized optic discs indicates that the performance and the localization are successful if the center of optic disc generated by the algorithm is within 60 pixels from the ground truth (C8) [19]. The evaluation procedures for both data sets are published with vessel detection algorithm and baseline results (C5) [18, 19].

DRIVE (digital retinal images for vessel extraction) [20, 21] is another well-known reference database for blood vessel detection (C3), which contains 40 retinal images (C6.2) with manually segmented pixel-wise ground truth (C2, C6.2). The manual segmentation task was divided between three medical experts, and the database was published along with vessel detection algorithm (C5) [21]. The detection performance is measured similarly as in the STARE database, that is, comparing the sensitivity to the specificity (C8.1) from which the area under curve (AUC) is computed to produce the final measure for the algorithm comparison (C8.2) [20, 21]. In addition, the authors implemented and internally evaluated a number of blood vessel detection algorithms from various research groups and the results were published in [22] and on the DRIVE database website (C4) [20].

MESSIDOR (methods to evaluate segmentation and indexing techniques in the field of retinal ophthalmology) [23] is a reference image database collected to facilitate computer-assisted image analysis of diabetic retinopathy. Its primary objectives are to enable evaluation and comparison of algorithms for analyzing the severity of diabetic retinopathy, prediction of the risk of macular oedema, and indexing and managing image databases, that is, support image retrieval. For the evaluation, the MESSIDOR database website [23] provides 1200 images (C6.2) with image-wise severity grading (C2, C6.2) from three ophthalmologic departments including descriptions for the severity grading. It is noteworthy to mention that the severity grading is based on the existence and number of diabetic lesions and their distance from the macula.

CMIF (collection of multispectral images of the fundus) [24, 25] is a public multispectral retinal image database. The spectral images were obtained by implementing a “filter wheel” into a fundus camera containing a set of narrow-band filters corresponding to the set of desired wavelengths [25]. The database itself consists of normal and abnormal images (C6.2) spanning a variety of ethnic backgrounds covering 35 subjects in total [25]. As such, the database is not ready for benchmarking, but it provides a new insight into retinal pathologies.

ROC (retinopathy online challenge) [26, 27] follows the idea of asynchronous online algorithm comparison proposed by Scharstein and Szeliski [28] for stereo correspondence algorithms (Middlebury Stereo Vision Page), where a web evaluation interface with public evaluation data sets ensures that the submitted results are comparable. The research groups download the data set, they submit their results in the required format, and the results are evaluated by the web evaluation system. Since the evaluation is fully automatic, the research groups can submit and update their results continuously. In the current state, the ROC database website [26] provides 100 retinal images (C6.2), a ground truth (C2, C6.2) and an online evaluation system for microaneurysms, and the evaluation results for a number of detection algorithms (C4). The algorithm performance is measured by comparing the sensitivity (the proportion of correctly classified lesions) against the average number of false positives in the image, that is, free-response receiver operating characteristic curve (FROC) (C8.1) [27]. The sensitivities of predefined false positive points are averaged to generate the final measure for algorithm comparison (C8.2) [27]. The annotations were gathered from 4 medical experts by marking the location, approximate size, and confidence of the annotation. Consensus of two medical experts was required for a lesion to be selected to the ground truth.

REVIEW (retinal vessel image set for estimation of widths) [29, 30] is a new reference image database to assess the performance of blood vessel width measurement algorithms. To characterize the different vessel properties encountered in the retinal images, the database consists of four image sets: (1) high-resolution image set (4 images); (2) vascular disease image set (8 images); (3) central light reflex image set (2 images), and (4) kick point image set (2 images) (C6.2). The REVIEW database concentrates on high-precision annotations, and, therefore, it provides only segments of blood vessels and not the whole network. To achieve high precision, the human observers used a semiautomatic tool to annotate a series of image locations from which the vessel widths were automatically determined [30]. The annotations were gathered from three medical experts, and the mean vessel width was defined as the ground truth (C2, C6.2). In the evaluation, the performance is measured using an unbiased standard deviation of the width difference between the algorithm-estimated vessel widths and the ground truth (C8) [30].

In general, most of the reference databases reach the minimal requirements for benchmarking image analysis algorithms; that is, they provide true patient images, ground truth from experts, and an evaluation protocol (Table 1). In some cases, the usability is already at a mature level, for example, in the case of the web evaluation system in the ROC database. The primary shortcomings appear to be related to the availability of software (C6.1) and how the algorithm's design for the medical problem is observed (C7). By publishing source codes or an executable, other researchers can avoid laborious reimplementation and if the database is correctly built to reflect real-world conditions, then the database implicitly reflects the applicability of the algorithm's design to the problem. The database properties in terms of the key questions are summarized in Table 1 and for comparison the proposed DiaRetDB1 database properties are summarized in Table 2. The framework for constructing benchmark databases and protocols has been summarized in Figure 1. The details of the framework are discussed in the next sections.

## 3. Patient Images and Ground Truth

### 3.1. Collecting Patient Images

The task of capturing and selecting patient images should be conducted by medical doctors or others specifically trained for photographing the eye fundus. With the images, there are two issues which should be justified: (1) distribution correspondence with the desired population and (2) privacy protection of patient data.

In DiaRetDB1, the ophthalmologists wanted to investigate the accuracy of automatic methods analyzing retinal images of patients who are diagnosed with having diabetes. Consequently, the images do not correspond to the actual severity or prevalence of diabetic retinopathy in the Finnish population but provide clear findings for automated detection methods. The data is, however, clinically relevant since the studied subpopulation is routinely screened by Finnish primary health care.

The privacy protection of patient data is a task related to the ethics of clinical practice, medical research, and also data security. A permission for collecting and publishing the data must be acquired from a corresponding national organization (e.g., national or institutional ethical committee) and from the patients themselves. Moreover, all data must be securely stored; that is, all patient information, such as identifying metadata, must be explicitly removed from images which are to be used in a public database. In DiaRetDB1, the retinal images were acquired using a standard fundus camera and its accompanying software. The acquired images were converted to raw bitmaps and then saved to portable network graphics (PNG) format using lossless compression. The raw bitmaps contained nothing but the pixel data which guaranteed the removal of hidden metadata.

### 3.2. Image Annotations as the Ground Truth

In general, the image annotations are essential for training supervised algorithms, as well as for their evaluation and comparison. Such information is typically collected by manually annotating a set of images. In face recognition, for example, a ground truth contains identifiers of persons in the images and often also the locations of facial landmarks, such as eye centers, which can be very useful in training the methods. Commonly, simple tailored tools are used to collect the data, but also generic applications are available for problems which require an exhaustive amount of image data, for example, LabelMe [31] Web tool for annotating visual object categories. Annotating medical images is not an exception, but two essential considerations apply: (1) annotations must be performed by clinically qualified persons (specialized or specializing medical doctors, or other trained professionals for specific tasks), denoted as “experts” and (2) the ground truth should include annotations from multiple experts.

A more technical problem is to develop a reusable tool for the annotation task. To avoid biasing the results, the experts should be given minimal guidance for their actual annotation work. Basic image manipulation, such as zoom and brightness control, for viewing the images is needed, and a set of geometric primitives are provided for making the spatial annotations. In LabelMe [31], the only primitive is polygon region defined by an ordered set of points. A polygon can represent an arbitrarily complex spatial structure, but ophthalmologists found also the following primitives useful: small circle, which can be quickly put on a small lesion, and circle area and ellipse area which are described by their centroid, radius/radii, and orientation (ellipse). The system also requires at least one representative point for each lesion. This point should represent the most salient cue, such as color or texture, that describes the specific lesion. Furthermore, a confidence selection from the set of three discrete values, low, moderate, or high, is required for every annotation. The experts are allowed to freely define the types of annotations, that is, the class labels for the lesion types, but typically it is preferable to agree with the labels beforehand (e.g., in DiaRetDB1: hard exudates, soft exudates, microaneurysms, and haemorrhages). An important design choice is related to the usability of the tool with respect to its graphical user interface (GUI). For example, the GUI should not use colors which distract the annotators from image content.

**Figure 2:**
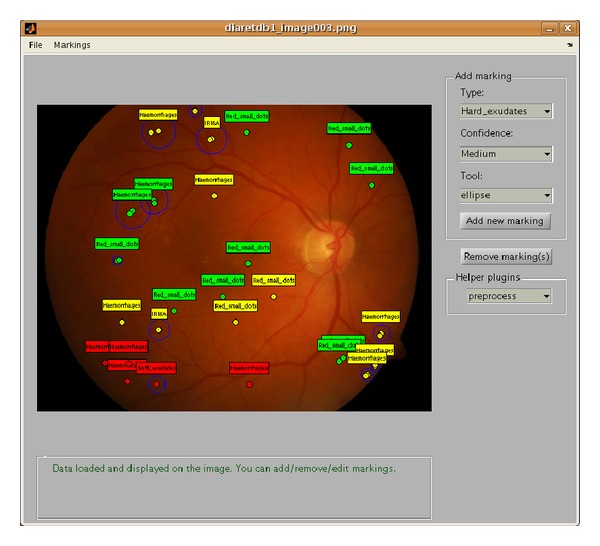
Graphical user interface of the image annotation tool [1].

The development of an annotation tool may take undesirable amount of research time and resources. To help other researchers in this task the tool is available upon request as Matlab M-files and as a Windows executable. Users have full access to the source code which enables tailoring of the tool for their specific needs. The default graphical user interface (GUI) is shown in Figure 2.

### 3.3. Data Format for Medical Annotations

To store the annotations and to be able to restore their graphical layout, the data format must be defined. The data is naturally structured, and, therefore, structural data description languages are preferred. Several protocols for describing medical data exist, such as HL7 based on the extensible markup language (XML) [32], but these are complex protocols designed for patient information exchange between organizations and information systems. Since the requirements for benchmarking databases in general are considerably less comprehensive, a light-weight data format based on the XML data description language is adopted. Instead of the XML Schema document description, a more compact and, consequently, more interpretable Document Type Definition (DTD) description is applied. The used format is given in Listing 1.

### 3.4. Fusion of Manual Segmentations from Multiple Experts

 A desired characteristic of collecting the ground truth for medical images is that one or several experts provide information on the image contents such as the disease-related lesions. Since there can exist inconsistencies in the case of a single expert (e.g., due to changing criteria while performing the annotation work) and nobody can be considered as the unparalleled expert, the use of several experts is preferred. Only in clear cases, however, the experts fully agree on the interpretation of the visible information. Since the early signs of retinopathy are very subtle changes in the images, it is necessary to develop a method to appropriately combine the expert information which is only partially coherent. To design such a method, the important questions relevant to training, evaluating, and benchmarking by using the database are as follows: (1) how to resolve inconsistencies in the annotations from a single expert and (2) how to fuse equally trustworthy (no prior information on the superiority of the experts related to the task) information from multiple experts?

**Figure 3:**
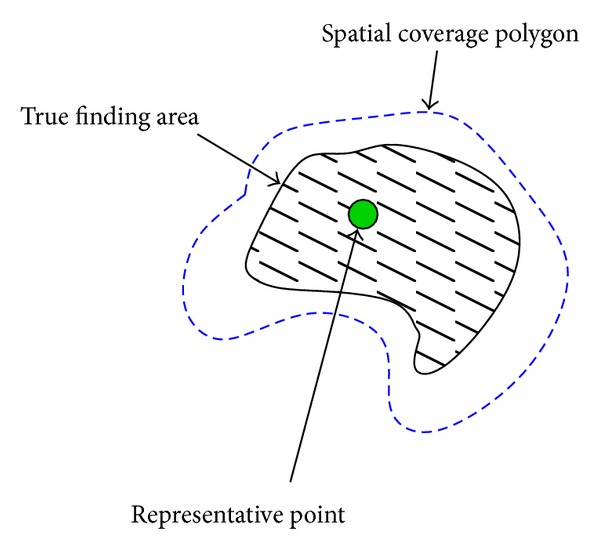
The available expert information in the DiaRetDB1 database. The expert's subjective confidence for the annotation is defined as follows: 100%, >50%, and <50% [1].

**Figure 4:**
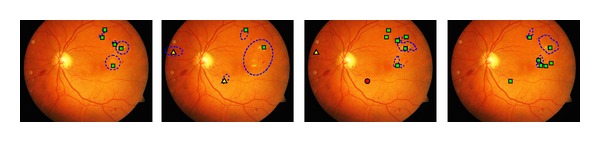
Four independent sets of spatial annotations (contours and representative points) for the same lesion type (hard exudates). The representative point markers denote the confidence level (*square* = 100%, *triangle* > 50%, and *circle* < 50%) [1].

In our data format, the available expert information is the following (Figure 3): (1) spatial coverage (polygon area), (2) representative point(s) (small circle areas), and (3) the subjective confidence level. The representative points are distinctive “cue locations” that attracted the expert's attention to the specific lesion. The confidence level with a three-value scale describes the expert's subjective confidence for the lesion to represent a specific class (lesion type) as shown in Figure 4.

Combining the manual segmentations from multiple experts was originally studied in [9]. In the study, the area intersection provided the best fusion results in all experimental setups and is computed in a straightforward manner as the sum of expert-annotated confidence images divided by the number of experts. For DiaRetDB1, the fused confidence with the threshold 0.75 yielded the best results [1], resolving the inconsistencies of annotations either from a single expert or multiple expert cofusion problems.

The area intersection is intuitive and the result is based on processing the whole image ensemble. However, the threshold was selected with the baseline method, which undesirably tied the training and evaluation together. Therefore, the combination problem was revised in [8].

**Figure 5:**
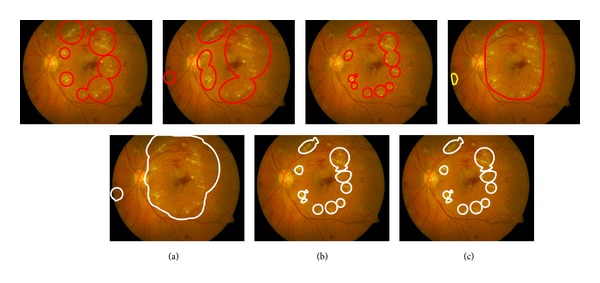
1st row: DiaRetDB1 expert spatial annotations for the lesion Hard exudate (red: high confidence, yellow: moderate, green: low). 2nd row: the ground truth (white) produced by the original method and (a) minimal and (b) maximal confidence. The disambiguated ground truth by (c) the revised method [8].

The most straightforward combination procedure is averaging where the expert segmentations are spatially averaged for each image and lesion type. In this procedure, the given confidence levels are used, and the only requirement for the confidence scale is that it is monotonically increasing. The average confidence image corresponds to the mean expert opinion, but it has two disadvantages: (1) it does not take into account the possible differences of the experts in their use of the scale and (2) it does not produce binary values for the foreground (lesion of specific type) and background. As a solution, a binary mask can be generated by thresholding the average expert segmentation image. The threshold parameter *τ* ∈ [0,1] adjusts experts' joint agreement: for *τ* → 0, the binary mask approaches *set union* and for *τ* → 1 approaches* set intersection* (see Figure 5).

The revised combining method is based on the following principle: *The ground truth should optimally represent the mutual agreement of all experts*. To evaluate the degree of mutual agreement, a performance measure is needed. The performance depends only on two factors: *experts' markings* and the *ground truth*, and, without loss of generality, the measure is expected to output a real number

(1) $$\begin{array}{c} \text{perf:}\left\{I_{\text{ex}{\text{p}}_{i,j,n}},g_{t_{i,j}}\right\}\to \mathbb{R}, \end{array}$$

where expert segmentation masks *I*_exp_*i*,*j*,*n*__ represents the expert segmentation mask for the input image *i*, lesion type *j*, and expert *n*, *g*_*t*_ is the ground truth, and {·} is used to denote that the performance is computed for a set of rated images. Generation of the image-wise ground truth is straightforward: if any of the pixels in the produced *I*_mask_*i*,*j*__ for the lesion *j* is nonzero, the image is labeled to contain that lesion. A detection ROC curve can be automatically computed from the image-wise ground truth and image scores computed from the expert images. For the image-wise expert scores, we adopted the summax rule described in Section 4: pixel confidences of *I*_exp_*i*,*j*,*n*__ are sorted, and 1% of the highest values are summed. The average equal error rate (EER point on the ROC curve) was chosen as the performance measure in (1), which can be given in an explicit form:

(2) perf({Iexpi,j,n},{gti,j}) =1N∑nEER({summax1%(Iexpi,j,n)},{Imaski,j(x,y;τ)}).

A single EER value is computed for each expert *n* and over all images (*i*), and then the expert-specific EER values are summed for the lesion type *j*.

The utilization of the summax rule is justified as a robust maximum rule by the multiple classifier theory [33]. Also the EER measure can be replaced with any other measure if, for example, prior information on the decision-related costs is available. The only factor affecting the performance in (2) is the threshold *τ* which is used to produce the ground truth. To maximize the mutual agreement, it is necessary to seek the most appropriate threshold $\hat{\tau }$ providing the highest average performance (EER) over all experts. Instead of a single threshold, lesion-specific thresholds ${\hat{\tau }}_{j}$ are determined since different lesions may significantly differ by their visual detectability. The optimal ground truth is equivalent to searching the optimal threshold:

(3) $$\begin{array}{c} {\hat{\tau }}_{j}⟵\underset{\tau_{j}}{\text{argmin}}\frac{1}{N}\underset{n}{\sum }\text{EER}\left(\cdot ,\cdot \right). \end{array}$$

A straightforward approach to implement the optimization is to iteratively test all possible values of *τ* from 0 to 1. Equation (3) maximizes the performance for each lesion type over all experts (*N*). The optimal thresholds ${\hat{\tau }}_{j}$ are guaranteed to produce the maximal mutual expert agreement according to the performance measure perf.

The revised combining method was shown to produce better results when compared to the original method and even to simultaneous truth and performance level estimation (STAPLE) [34]. The full description of the method and comparisons is presented in [8].

## 4. Algorithm Evaluation

### 4.1. Evaluation Methodology

The ROC-based analysis perfectly suits to medical decision making, being the acknowledged methodology in medical research [35]. An evaluation protocol based on the ROC analysis was proposed in [6] for image-based (patient-wise) evaluation and benchmarking, and the protocol was further studied in [9]. In clinical medicine, the terms *sensitivity* and *specificity* defined in the range [0%, 100%] or [0,1] are used to compare methods and laboratory assessments. The sensitivity

(4) $$\begin{array}{c} \text{SN}=\frac{\text{TP}}{\text{TP}+\text{FN}} \end{array}$$

depends on the diseased population whereas the specificity

(5) $$\begin{array}{c} \text{SP}=\frac{\text{TN}}{\text{TN}+\text{FP}} \end{array}$$

on the healthy population, defined by true positive (TP), true negative (TN), false positive (FP), and false negative (FN). The *x*-axis of an ROC curve is 1 − specificity, whereas the *y*-axis represents directly the sensitivity [12].

It is useful to form an ROC-based quality measure. the quality measures preferred are as follows: The equal error rate (EER) [36] defined as when (SN = SP)

(6) $$\begin{array}{c} \text{SN}=\text{SP}=1-\text{EER}, \end{array}$$

or weighted error rate (WER) [37]

(7) $$\begin{array}{c} \text{WER}\left(\hat{R}\right)=\frac{\text{FPR}+\hat{R}\cdot \text{FNR}}{1+\hat{R}}=\frac{\left(1-\text{SP}\right)+\hat{R}\cdot \left(1-\text{SN}\right)}{1+\hat{R}}, \end{array}$$

where $\hat{R}=C_{\text{FNR}}/C_{\text{FPR}}$ is the cost ratio between the false negative rate FNR = 1 − SN = FN/(TP + FN) and false positive rate FPR = 1 − SP = FP/(FP + TN). The main difference between the two measures is that EER assumes equal penalties for both false positives and negatives, whereas in the WER, the penalties are adjustable.

**Figure 6:**
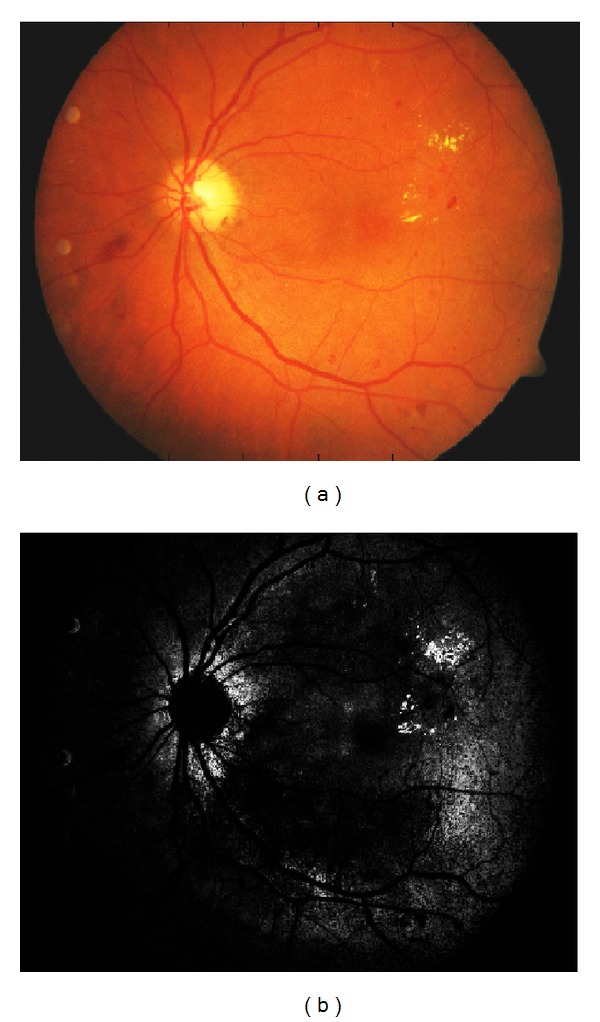
Pixel-wise likelihoods for Hard exudates produced by the strawman algorithm: (a) original image (hard exudates are the small yellow spots in the right part of the image); (b) “likelihood map” for hard exudates [9].

In the image-based evaluation, a single likelihood value for each lesion should be produced for all test images. Using the likelihood values, an ROC curve can be automatically computed [9]. If a method provides multiple values for a single image, such as the full-image likelihood map in Figure 6(b), the values must be fused to produce a single score.

### 4.2. Image-Based Evaluation

The automatic image-based evaluation follows the medical practice where the decisions are “subject-wise.” An image analysis system is treated as a black-box which takes an image as the input. If the images are assumed to be either normal or abnormal, the system produces a score that corresponds to the probability of the image being abnormal, and a high score corresponds with high probability. The objective of the image-based evaluation protocol is to generate an ROC curve by manipulating the score values of the test images. The practices were adopted from [38].

Let the image analysis algorithm produced score values for *n* test images be **ζ** ^im^ = {*ζ*_1_^im^,…, *ζ*_*n*_^im^} and let the corresponding image-wise ground truths be **ω** ^im^ = {*ω*_1_^im^,…, *ω*_*n*_^im^}, where each *ω*_*i*_^im^ is either “normal” or “abnormal.” Then, by selecting a threshold for the score values (**ζ** ^im^), the test images can be classified as either normal or abnormal, and the performance expressed in the form of sensitivity and specificity can be determined by comparing the outcome with the corresponding image-wise ground truth (**ω** ^im^). If the same procedure is repeated using each test image score as the threshold, the ROC curve can be automatically determined since each threshold generates a (sensitivity, specificity) pair that is a point on the ROC curve. Consequently, the procedure requires that the test images include samples from both populations, normal and abnormal. The image score-based evaluation method is presented in Algorithm 1.

### 4.3. Pixel-Based Evaluation

To validate a design choice in method development, it can be useful to measure also the spatial accuracy, that is, whether the detected lesions are found in correct locations. Therefore, a pixel-based evaluation protocol which is analogous to the image-based evaluation is proposed. In this case, the image analysis system takes an image as the input and outputs a similar score for each pixel. The objective of the pixel-based evaluation is to generate an ROC curve which describes the pixel-level success.

Let the image analysis algorithm-produced pixel score values for all *n* pixels in test set be **ζ** ^pix^ = {*ζ*_1_^pix^,…, *ζ*_*n*_^pix^} and let the corresponding pixel-wise ground truth be **ω** ^pix^ = {*ω*_1_^pix^,…, *ω*_*n*_^pix^}, where the **ω** ^pix^ is either “normal” or “abnormal.” Then, by selecting a global pixel-wise threshold for the pixel score values (**ζ** ^pix^), the pixels in all images can be classified to either normal or abnormal. Now, the sensitivity and specificity can be computed by comparing the outcome to the pixel-wise ground truth (**ω** ^pix^). If the procedure is repeated using each unique pixel score as the threshold, the ROC curve can be automatically determined. The pixel-wise evaluation procedure is given in Algorithm 2. Note that the abnormal test image pixels contribute to both sensitivity and specificity, whereas the normal images only contribute to the specificity.

The evaluation forms a list of global pixel-wise scores from the test image pixel scores which determines the score thresholds. The use of all unique pixel scores in the test images is time consuming if the number of images in the test set is large or high-resolution images are used. The problem can be overcome by sampling the test image pixel scores. To preserve the test set's pixel score distribution, the global threshold scores can be devised as follows: (1) sort all the unique pixel scores in an ascending order to form an ordered sequence *L* and (2) compose the new reduced sequence of pixel scores *L*_sampled_ by selecting every *j*th likelihood in *L*.

### 4.4. The Strawman Algorithm

 We provide a baseline method in the form of a strawman algorithm. The algorithm is based on the use of photometric cue as described in Algorithm 3 [9].

The score fusion in the strawman algorithm is based on the following reasoning: if we consider *M* medical evidence (features) extracted from the image, **x**_1_,…, **x**_*M*_, where each evidence is a vector, then we can denote the score value of the image as *p*(**x**_1_,…, **x**_*M*_ | abnormal). The joint probability is approximated from the classification results (likelihoods) in terms of decision rules using the combined classifier theory (classifier ensembles) [33]. The decision rules for deriving the score were compared in the study [9] where the rules were devised based on Kittler et al. [33] and an intuitive rank-order-based rule “summax.” The rule defines the image score *p*(**x**_1_,…, **x**_*M*_ | abnormal) using the compared decision rules when the prior values of the population characteristics are equal (*P*(normal) = *P*(abnormal)) as follows:

(8) $$\begin{array}{c} \text{SCOR}{\text{E}}_{\text{summax}}=\underset{m\in N_{Y\%}}{\sum }p\left(x_{m}\;|\;\text{abnormal}\right), \end{array}$$

where *N*_*Y*%_ are the indices of *Y*% top-scoring pixel scores. Experimenting also with the max, mean, and product rules, strong empirical evidence supports the rank-order-based sum of maxima (summax; proportion fixed to 1%) [9]. 

**Figure 7:**
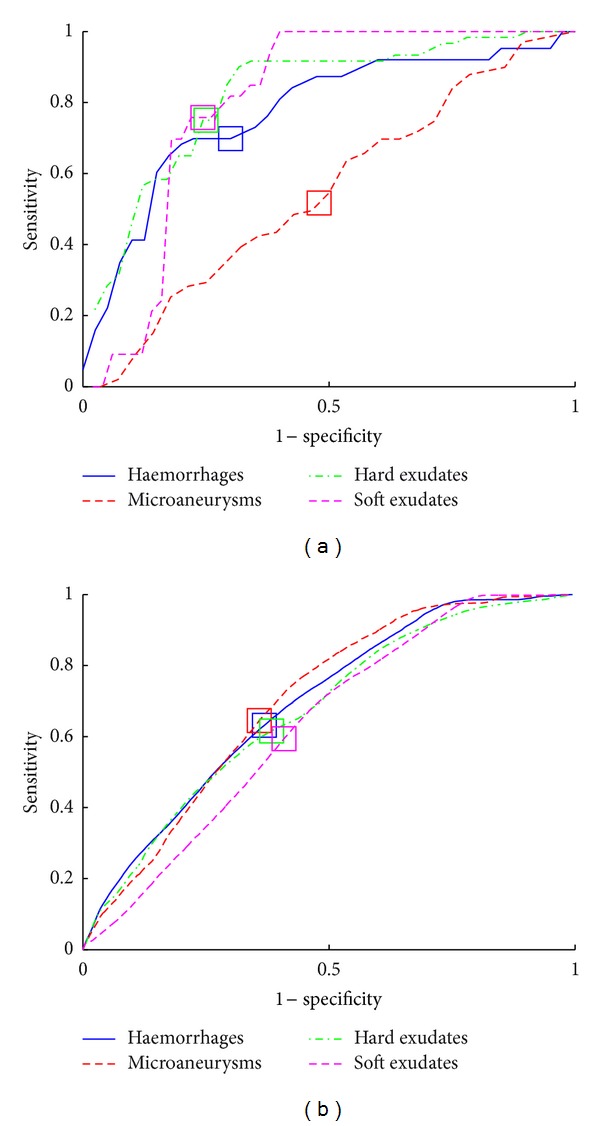
The ROC curves for the DiaRetDB1 strawman algorithm using the original ground truth (*squares* denote the EER points): (a) image based; (b) pixel based. Note the clear difference with microaneurysms as compared to the revised ground truth in Figure 8.

The achieved results for DiaRetDB1 are shown in Figure 7 (ROC curves) and in Table 3 (EER values). The performance is reported by using the EER which is justified since EER represents a “balanced error point” on the ROC curve and allows comparison to the previous works.

To quantify the effect of the revised method for combining the expert information, results from a comparison are shown in Table 3. It should be noted that the experiment is independent of the one presented above. The original confidence threshold (0.75) in [9] was not optimal for any of the lesion types and was clearly incorrect for haemorrhages (HA, 0.60) and microaneurysms (MA, 0.10). The underlined values in the table are the best achieved performances. The average performance for all lesion types significantly varies depending on the threshold.

**Figure 8:**
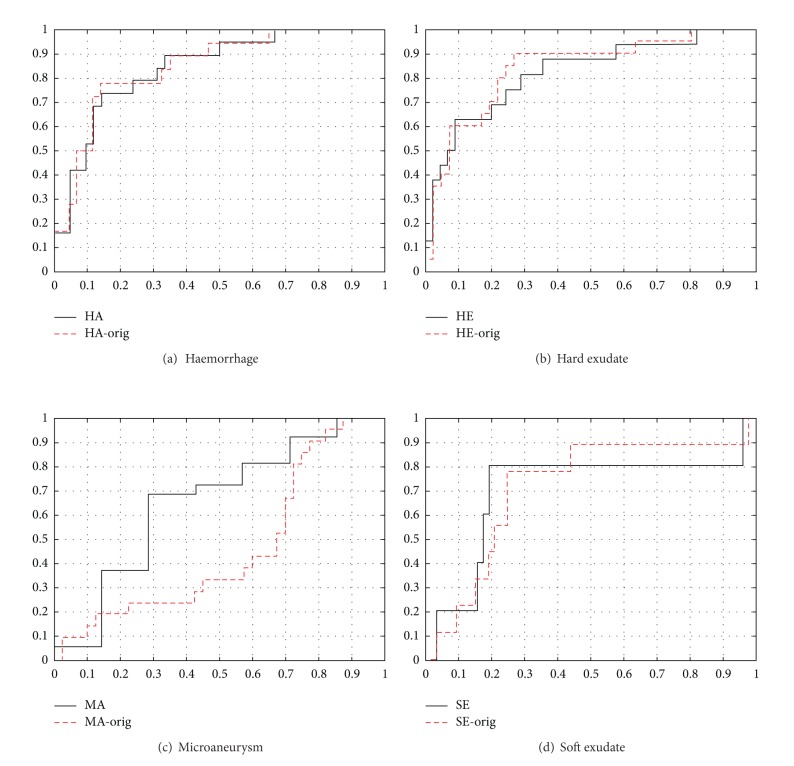
ROC curves for the DiaRetDB1 baseline method using the original and revised (max) method to generate the training and testing data [8].

The minimum and maximum thresholds for the revised combining method produce equal results except in the case of soft exudates, for which the maximum in the equally performing interval (1.0) is clearly better. The main difference from the original DiaRetDB1 method occurs with microaneurysms, since the optimal threshold (0.1) significantly differs from the original (0.75). For haemorrhages, the original result was too optimistic since the optimal confidence yields worse minimum and average EER. On average, the revised method provided 11–17% better performance. The related ROC curves are shown in Figure 8. 

## 5. Case Study: DiaRetDB1 Diabetic Retinopathy Database and Protocol V2.1

**Figure 9:**
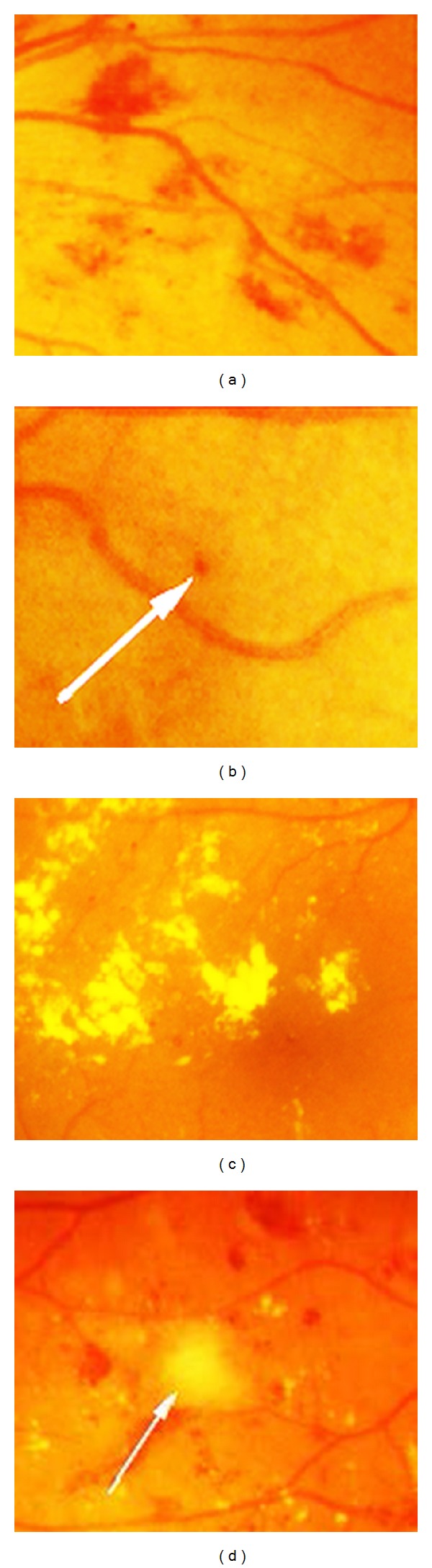
Abnormal retinal findings caused by the diabetes (best viewed in colour): (a) haemorrhages; (b) microaneurysms (marked with an arrow); (c) hard exudates; (d) soft exudate (marked with an arrow) [6].

The authors have published two medical image databases with the accompanied ground truth: DiaRetDB0 and DiaRetDB1. The work on DiaRetDB0 provided us with essential information on how diabetic retinopathy data should be collected, stored, annotated, and distributed. DiaRetDB1 was a continuation to establish a better database for algorithm evaluation. DiaRetDB1 contains retinal images selected by experienced ophthalmologists. The lesion types of interest were selected by the medical doctors (see Figure 9): microaneurysms (distensions in the capillary), haemorrhages (caused by ruptured or permeable capillaries), hard exudates (leaking lipid formations), soft exudates (microinfarcts), and neovascularisation (new fragile blood vessels). These lesions are signs of mild, moderate, and severe diabetic retinopathy, and they provide evidence also for the early diagnosis. The images were annotated by four independent and experienced medical doctors inspecting similar images in their regular work. 

The images and ground truth are publicly available on the Internet [13]. The images are in PNG format, and the ground truth annotations follow the XML format. Moreover, we provide a DiaRetDB1 kit containing full Matlab functionality (M-files) for reading and writing the images and ground truth, fusing expert annotations, and generating image-based evaluation scores. The whole pipeline from images to evaluation results (including the strawman algorithm) can be tested using the provided functionality. The annotation software (Matlab files and executables) is also available upon request.

## 6. Conclusions

 We have discussed the problem of establishing benchmark databases for the development of medical image analysis. We have pointed out the importance of commonly accepted and used databases. We have proposed the framework for constructing benchmark databases and protocols for diabetic retinopathy in medical image analysis. We have built reusable tools needed to solve the important subtasks, including the annotation tool for collecting the expert knowledge, made our implementations publicly available, and established the diabetic retinopathy database DiaRetDB1 to promote and help other researchers collect and publish their data. We believe that public databases and common evaluation procedures support development of better methods and promote the best methods to be adopted in clinical practice.

## Figures and Tables

**Algorithm 1:**
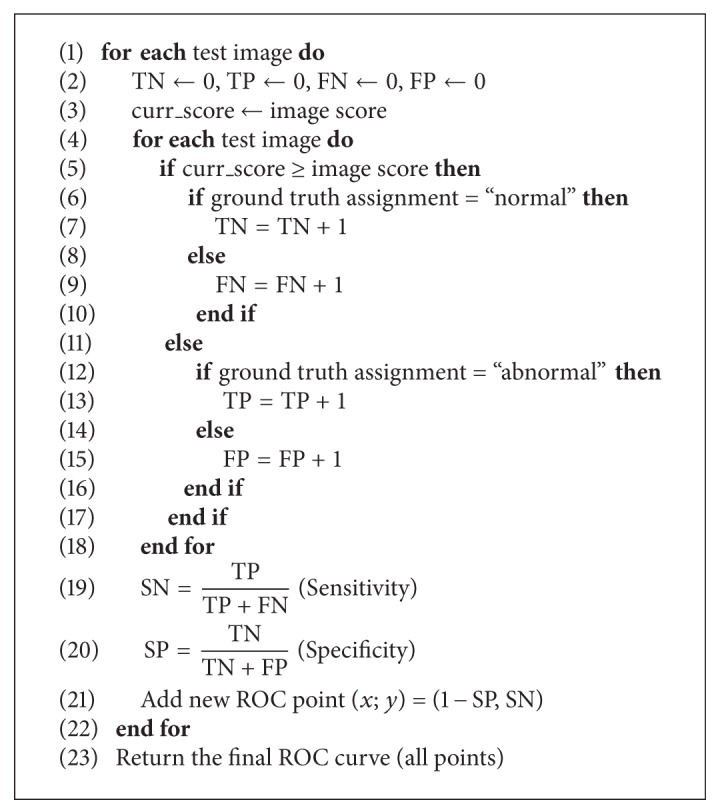
Image-wise evaluation based on image scores.

**Algorithm 2:**
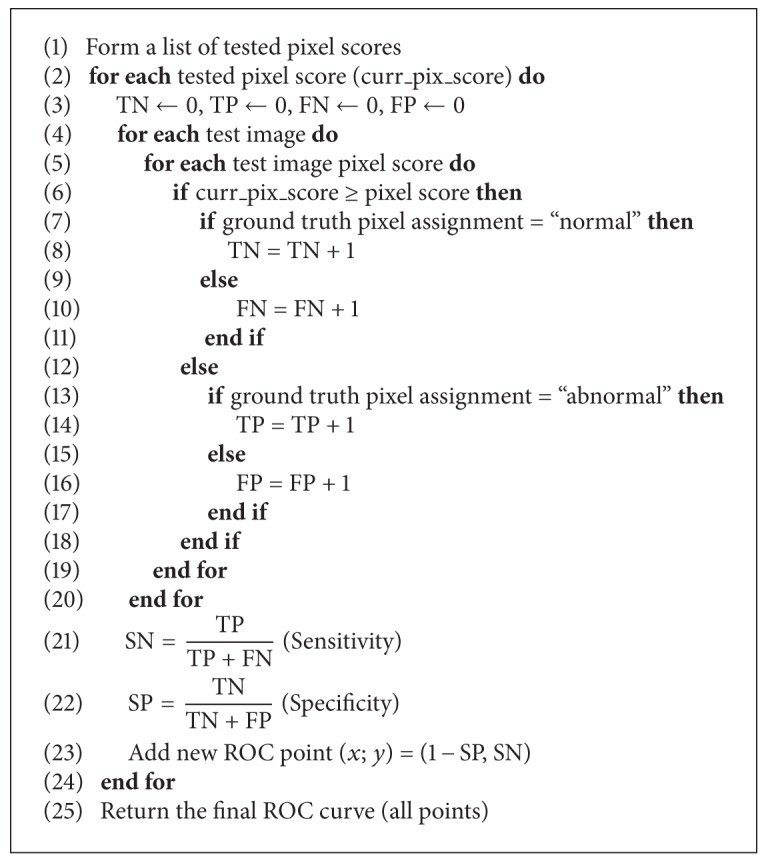
Pixel-wise evaluation based on pixel scores.

**Algorithm 3:**
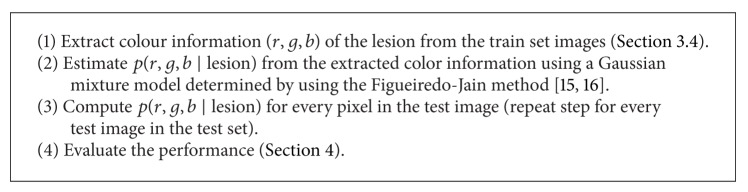
Strawman algorithm.

**Listing 1:**
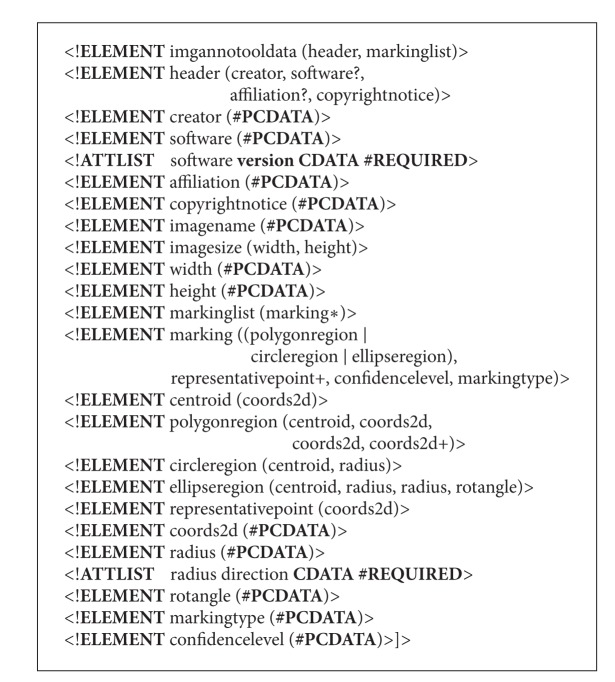
DTD definition.

**Table 1:** Summary of the current state of the reference image databases in terms of the key questions addressed in Section 2.1.

Key questions	STARE (vessel)	STARE (disc)	DRIVE	MESSIDOR	CMIF	ROC	REVIEW
C2: “Is there a data set for which the correct answers are known?”	x		x	x		x	x
C3: “Are there data sets in common use?”	x	x	x	x	x	x	x
C4: “Are there experiments which show algorithms are stable and work as expected?”	x		x			x	
C5: “Are there any strawman algorithms?”	x	x	x				
C6.1: “What code is available?”						x	
C6.2: “What data is available?”	x	x	x	x	x	x	x
C7: “Is there a quantitative methodology for the design of algorithms?”							
C8.1: “What should we be measuring to quantify performance?”	x	x	x			x	x
C8.2: “What metrics are used?”		x	x			x	x

∑‍	6	5	7	3	2	7	5

**Table 2:** Summary of the DiaRetDB1 V2.1 database in terms of the key questions addressed in Section 2.1.

Key questions	DiaRetDB1 V2.1
C2: “Is there a data set for which the correct answers are known?”	Yes
C3: “Are there data sets in common use?”	Yes (publicly available at [13])
C4: “Are there experiments which show algorithms are stable and work as expected?”	Experimental results reported in Section 4.4
C5: “Are there any strawman algorithms?”	No, but the baseline algorithm sets the baseline results for the DiaRetDB1 database
C6.1: “What code is available?”	Functionality for reading/writing images and ground truth, strawman algorithm, and annotation software (publicly available at [13, 14])
C6.2: “What data is available?”	Images and ground truth (XML) (publicly available at [13])
C7: “Is there a quantitative methodology for the design of algorithms?”	No, but medical practice is used as a guideline at each development step
C8.1: “What should we be measuring to quantify performance?”	Image- and pixel-based ROC analysis (description in Section 4)
C8.2: “What metrics are used?”	Equal error rate (EER) defined in Section 4

**Table 3:** The minimum, maximum, and average EER (5 random iterations) for the baseline method and evaluation protocol when using DiaRetDB1. The results include the original and the revised ground truth [8].

	Haemorrhage (HA)			Hard exud. (HE)			Microaneurysm (MA)			Soft exud. (SE)			Overall
	Min	Max	Avg	Min	Max	Avg	Min	Max	Avg	Min	Max	Avg	
In [9]	0.233	0.333	0.273	0.200	0.220	0.216	0.476	0.625	0.593	0.250	0.333	0.317	0.349
In [8] (min)	0.263	0.476	0.322	0.250	0.250	0.250	0.286	0.574	0.338	0.333	0.333	0.333	0.311
In [8] ( max)	0.263	0.476	0.322	0.250	0.250	0.250	0.386	0.574	0.338	0.200	0.268	0.241	**0.288**
